# Use of the step-up action research model to improve trauma-related nursing educational practice

**DOI:** 10.4102/curationis.v38i2.1493

**Published:** 2015-10-23

**Authors:** Ielse Seale, Johanna C. de Villiers

**Affiliations:** 1 School of Nursing, University of the Free State, South Africa

## Abstract

**Background:**

A lack of authentic learning opportunities influence the quality of emergency training of nursing students. The purpose of this article is to describe how the step-up action research model was used to improve the quality of trauma-related educational practice of undergraduate nursing students.

**Objectives:**

To reduce deaths caused by trauma, healthcare workers should be competent to provide emergency care and collaborate effectively with one another.

**Method:**

A simulated mass casualty incident, structured to support the integration of theory into practice, became a more rigorous action research activity which focused on the quality improvement of the mass casualty incident.

**Results:**

The results indicated improved student learning; partnership appreciation; improved student coping mechanisms, and increased student exposure. Quality emergency training thus results in better real-life collaboration in emergency contexts.

**Conclusion:**

The step-up action research model proved to be a collaborative and flexible process. To improve the quality and rigour of educational programmes it is therefore recommended that the step-up action research model be routinely used in the execution of educational practices.

## Introduction

The statistics of people seriously injured and dying in motor vehicle accidents are high worldwide. According to a Global status report on road safety 2013, ‘road accidents kill more than 1,24 million people annually’ (WHO 2013). How many of these deaths could have been prevented by immediate effective interventions? Hogan and Boone (2008:681) point to what is known as the ‘golden hour’ in trauma intervention. This concept arose from French casualty data during the First World War, where patients treated within one hour after sustaining an injury had a 10% mortality rate, whereas those treated eight hours after an injury had a 75% mortality rate. Early scene interventions where patients are classified according to the severity of their injuries are the most important medical function at a mass casualty incident (MCI) (Garner *et al.* 2001:541). To reduce deaths caused by trauma, healthcare workers should be competent to provide emergency care and effectively collaborate with each other, especially during the golden hour. The researchers therefore realise that patient outcomes could be enhanced when healthcare workers understand the dynamics of collaboration with each other in trauma-related situations.

Green and Purvis (2004:14) state that opportunities for the placement of undergraduate students in emergency-care settings are limited. This lack of opportunities might influence the quality of their training, making simulation an option worth considering. Onda (2011:e273, e274, e277) states that simulation is a valuable teaching and learning strategy that promotes situated learning, clinical reasoning and problem solving and provides realistic environments. Simulation therefore provides an efficacious alternative opportunity for learning in an authentic environment. It is an innovative teaching and learning strategy that can be used in almost all levels of nursing education to improve skills and critical thinking in a safe environment (Rothgeb 2008:493).

The purpose of this article is to describe how the step-up action research (AR) model (Seale, Wilkinson & Erasmus 2005:215) was used to improve the quality of trauma-related nursing educational practice within an undergraduate nursing programme, where students participate in a simulated MCI together with various healthcare workers.

The article commences with a situational context to provide readers with background information. This is followed by a statement of the paradigm and the methodological principles, and a description of the step-up AR model. The results of AR set one are discussed and brief mention of set two is made, after which the article is concluded.

### Situational context

At the School of Nursing, University of the Free State (UFS), the degree programme uses outcomes-based education (OBE), which is a student-centred teaching and learning approach. This approach is aligned to the constructivist principle of making meaning, where students rebuild their knowledge based on new experiences (Rodgers 2007:73). In OBE the aim is that students will gain knowledge, skills and attitudes in order to become lifelong learners and fulfil meaningful professional roles through the integration of theory and practice in real-life situations (Maree & Fraser 2004:4). Emergency care is included in the four-year professional degree programme (hereafter the degree) as a vertical curriculum strand where life-support practices are developed across all four years. The emergency care theme is presented by means of lectures, case studies, practical demonstrations and authentic simulations, and is assessed annually by means of written and practical examinations.

The MCI is simulated in the third year of the degree as one of the main learning strategies in addition to real-life exposure to integrate theory and practice. The first simulation was a taxi accident scene which took place on a busy road next to a male residence on the campus. There were many incidental onlookers such as students and campus personnel. Qualified emergency healthcare providers facilitated the scene. Half of the students simulated victims with injuries and the other half simulated emergency healthcare providers. The students simulating healthcare providers had to act on the scene upon arrival. Apart from the theoretical lecture in class, no guidance was given to the students regarding the MCI.

After this first MCI simulation, the facilitators of the School of Nursing reviewed and improved it annually. The review of the simulation became a more rigorous AR initiative to improve the quality of this trauma-related educational strategy. The paradigm and methodology of this initiative are addressed in the next section.

## Methods

This research was grounded in a phenomenological paradigm which assumes that knowledge can be created on the basis of personal and professional experience and reflection on this experience (Zuber-Skerritt & Fletcher 2007:423). AR within this paradigm was used as the research method. The classic description of AR includes aspects such as interlinked action and reflection to improve one’s own work situation, participation in decision-making and making experiences public (Altrichter *et al.* 2002:129, 130). Quality AR should meet certain principles, amongst which self-criticism, reflection and ethical implementation are imperative. AR should improve practice and include all stakeholders, including those who will be affected by the research. This research contributes to theoretical and practical knowledge within and across systems through rigour and innovation (Zuber-Skerritt & Fletcher 2007:417*)*.

### Population and sample

The population for the study consisted of all the participants in the MCI. The purposive sample consisted of three groups of people, which included all those who took part in the MCI: (1) the third-year nursing students (*N* = 29), (2) the faculty members involved in the third-year programme (*N* = 5) and (3) external partners, who comprised paramedics (*N* = 30), staff members of police force (*N* = 2), one traffic policeman, fire brigade personnel (*N* = 5), ambulance services (*N* = 10), defence force (*N* = 3) and protection services (*N* = 2) at the UFS.

Purposive sampling for AR set two took place the following year and also included the three groups of people who took part in the MCI of that year. The three groups were: (1) the third-year nursing students (*N* = 45), (2) the faculty members involved in the third-year programme (*N* = 5) and (3) external partners, who comprised paramedics (*N* = 25), staff members consisting of one police person, one traffic policeman, fire brigade members (*N* = 4), ambulance services personnel (*N* = 8), the hospital emergency unit staff (*N* = 6) and one protection services member at the UFS.

### Data collection

Data was collected by means of multiple sources and was classified according to the taxonomy given by Mills (2007:4, 49), namely, examining, experiencing and enquiring (refer Table 1). Quantitative data were collected from the students by using a questionnaire that served as the primary source. The questionnaire was peer reviewed by faculty members before it was used. Essential content of the questionnaire was identified through a brainstorming session attended by the faculty members (see student questionnaire column in Table 4). At this session, each staff member generated as many ideas as they could about the problem in question. To avoid misinterpretations of the data, open-ended responses as well as responses to set questions were included in the questionnaire. The open-ended responses improved the validity of the data by including the authenticity of the student voice (Botma *et al.* 2010:134).

Supportive qualitative data was collected from the rest of the population through minutes of meetings, reflective discussions and observation. The supportive data were triangulated with the primary data to improve the validity and rigour (Mertens 2010:298).

**TABLE 1:** Data collection methods used, classified according to Mills’ taxonomy.

Participants	Data collection sources	Mills’ data classification
Students	Minutes of meetings (faculty)	Examining
Faculty members	Observation (notes and reports of researchers)	Experiencing
External partners:	Reflective discussions (MCI debriefing)	
Paramedics, police, fire brigade, ambulance services, traffic police, defence force		-
Local hospitals, protection services at the UFS		
Students	Questionnaires	Enquiring

MCI, mass casualty incident; UFS, University of Free State.

### Data analysis

The quantitative data gathered were analysed by the faculty members. The data were entered into a spreadsheet, printed and checked. Descriptive statistics, namely frequencies and percentages, were calculated.

The five faculty members applied deductive interpretive reasoning during the analysis of the qualitative data. In accordance with the deductive process (Botma *et al.* 2010:194), suitable phrases or words that relate to the South African critical cross-field outcomes^1^ which seemed to be appropriate to the specific MCI context were searched. Consensus about categorising the data according to these outcomes was reached and a paper trail was kept, thus enhancing rigour.

Trustworthiness or gaining a true understanding of the data was achieved by four strategies (Botma *et al.* 2010:235). The credibility and dependability of the analysed data were ensured through member checking (with research participants) and peer debriefing (with other researchers) (Mertens 2010:257, 431). The data were described as clearly as possible to enable transferability in other contexts. Confirmability was addressed through triangulation. Data triangulation involved the inclusion of perceptions from all participants to strengthen the interpretation and ensure validity and rigour (Botma *et al.* 2010:235; Mertens 2010:429).

### Ethics

Ethical clearance was granted for the study by the Ethics Committee of the Faculty of Health Sciences (UFS). The principles for ethical practice (axiology) of qualitative research were followed as described by Mertens (2010:16–18) to ensure rigor with regard to privacy, informed consent, confidentiality, honesty, that no harm was done and that ethical standards were maintained. All the students had to take part in the MCI as part of their curriculum. In order to be included in the study they had to agree by means of a written consent to the publication of the research outcomes.

In the next section, the implementation of the step-up model is discussed.

### Step-up model in action: Set one

**FIGURE 1:**
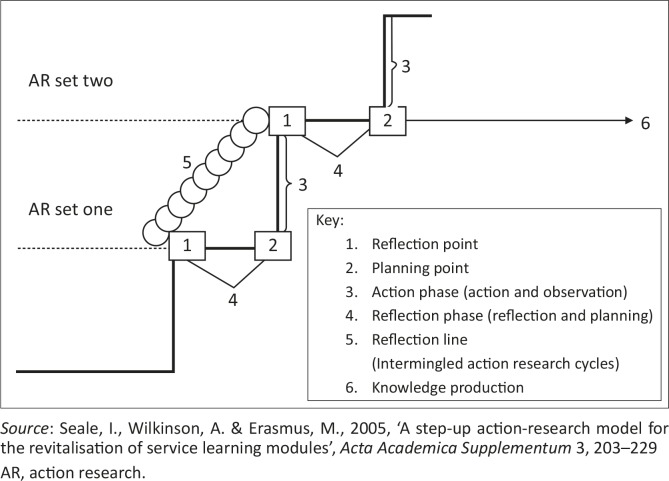
The step-up action research model

The AR spiral model consists of four phases: planning, acting, observing and reflecting. The step-up model (Figure 1) adapted from the spiral model consists of ascending AR steps, where each step represents an AR set or time period, each of which, in turn, consists of two phases, namely a reflection phase and an action phase (Seale *et al.* 2005:215).

In the first AR set (Figure 1), the faculty members involved in teaching the third-year students reflected on the simulated MCI and documented plans for new actions to be performed in the following two years. Seale *et al.* (2005:216) explain that, during the reflection phase of a set (Figure 1, number 4), reflection and planning are the forerunners of the action phase (Figure 1, number 3), where the planned actions will be implemented and data collected.

The selection and preparation of the actions for AR set one took place at the planning point of the step-up model (Figure 1, number 2) and initiated the departure of the action phase (Figure 1, number 3)*.* Observation (the process of data collection, analysis and interpretation), as part of the action phase, took place during and after the MCI and was documented carefully throughout.

Within each step-up AR set, multitudes of intermingled AR cycles occur to signify formative reflection. This is illustrated by using a diagonal line in the form of a spiral (Figure 1, number 5) that cuts through each reflection point (Figure 1, number 1). Perpendicular upward movement of the action phase represents quality improvement and change taking place during the implementation. Seale *et al.* (2005:215) explain that knowledge production is a result of the processes of reflection and innovative redesigning. The horizontal forward movement of the reflection phase to the right indicates the contribution to scholarly knowledge (Figure 1, number 6).

The goals and complementary actions of the MCI that the AR initiative focused on are represented in Table 2.

**TABLE 2:** Goals and the complementary actions of the mass casualty incident.

Goals	Actions
Improve student learning	1.Align experiences of MCI outcomes according to the critical cross-field outcomes
Promote partnership appreciation and collaboration	2. Form mixed teams of partners for the MCI implementation
Improve student coping mechanisms	3. Debrief and reflect after the MCI
Increase student exposure	4. Use smaller team groups with more casualties

MCI, mass casualty incident.

#### Actions of set one

The first action for AR set one was to align the learning experience of the MCI outcomes with the critical cross-field outcomes (Table 3). The simulated MCI was adapted to include more of the notions embedded in the critical cross-field outcomes.

**TABLE 3:** Critical cross-field outcomes align with learning experience of the mass casualty incident.

Learning experience related to the theme outcomes	Critical cross-field outcomes
Trauma patients triaged on the scene	Critical thinking and problem solving
	Managing of self
	Research and interpretation of information
	Working with others
Primary assessment of victims	Critical thinking and problem solving
	Managing of self
	Research and interpretation of information
Secondary assessment of victims	Critical thinking and problem solving
	Research and interpretation of information
	Working with others
Partnerships formed with relevant institutions	Working with others
	Managing of self
	Effective communication
	Use of science and technology
	Seeing world as a set of related systems
Students exposed to trauma victims	Critical thinking and problem solving
	Research and interpretation information
	Managing of self
	Research and interpretation of information
	Seeing world as a set of related systems

MCI, mass casualty incident.

The second action for AR set one was to form mixed teams of partners for the MCI implementation to promote partnership appreciation. The aim of this action was to foster professional respect between the nursing students and the emergency healthcare providers who assisted the students in order to promote inter-disciplinary collaboration. The importance of this aspect is recognised by, for example, Melby (2000:644), who states that the unique skills of other professionals are essential if collaborative relationships are to be formed.

The main partner was the Central University of Technology (CUT). The CUT offers a three-year diploma in Emergency Medical Care and Rescue designed for paramedics. In the CUT curriculum experiential learning is the principal teaching and learning approach. It was thus within these partnerships that shared implementation of the MCI took place.

The third action for AR set one, namely debriefing on the MCI to optimise coping behaviour, involved all the role-players. Rodgers (2007:15) describes debriefing as a means of encouraging students to reflect on action and a stimulus for them to consider how to change their actions in order to improve learning.

As part of action four of AR set one, smaller team groups with more casualties were used to increase student exposure. Groups of eight students were allocated to a final-year paramedic student, who acted as a facilitator for the group, instead of sending all the students into the simulation to participate at once without close guidance. The rationale was to broaden student learning opportunities and, at the same time, to have the students supported by the paramedics.

## Results, discussion and reflection

The results, discussion and reflection follow in the next section.

### Results and discussion of actions research set one

The critical cross-field outcomes served as broad themes for the data analysis of all four actions. Table 4 summarises the quantitative results of the 29 student questionnaires jointly, with the qualitative results of all the participants related to all four actions for AR set one.

**TABLE 4:** Results of action research set one according to the critical cross-field outcomes.

Critical cross-field outcomes	Student questionnaire: Results (*N *= 29)	Percentage	Qualitative direct quotes related: All partners
Critical thinking and problem solving Research and interpretation of information	Applied theory to MCI	83	‘need more knowledge’
	-	-	‘learn to work and think under pressure’
	Used learning acquired during MCI in real life later	89	‘learn how to sort people in disaster’
			‘prioritisation’
			‘made me understand what happened in an actual scene’
			‘eye-opener for real life’
Use of science and technology	Effective use of medical equipment	96	
Seeing world as a set of related systems	Clarity of roles:		‘understand roles better’
	1. Paramedic	89	‘realise important role of everyone’
	2. Triage officer	96	‘being a paramedic is not that simple’
	3. Transport officer	93	‘take them seriously’
Working with others	Positive attitude change towards role-players	55	‘team work and not individual’
			‘Trained group leader helps to:
			• organise the whole group
			• set everybody at ease
			• manage the chaos better’
			‘exciting and learn a lot’
			‘respect for them [*paramedics*]’
			‘honour paramedics’
Managing of self	Not applicable	Not applicable	‘felt stupid and discouraged because not trained’
			‘motivated to do things right and efficiently to save lives’
			‘you have to be fast’
			‘do it as fast as possible’
			‘do not panic’
			‘patience’
			‘realised [*I*] must stay focused’
Effective communication	Reflection on principles of triage during the experience	83	‘learn to communicate on the scene’
			‘Trained group leader helps to:
			• direct what needs to be done
			• give guidance
			• save time
			• give comments
			• get everything done perfectly
			• delegate tasks
			• plan ahead
			• advice
			• coordinate actions’
			‘Debriefing session:
			• helps to know what is right and wrong
			• is empowering
			• ‘helps to learn about shortcomings’
Discrete outcomes: Exploring education and career opportunities	Possible occupational options considered	48	‘this job needs somebody with passion and a spirit of saving other people’s lives’

*Student learning* was enhanced through structuring the MCI according to the critical cross-field outcomes (action 1). Most students (83%) said they could apply theory to practice reflecting their critical thinking and problem-solving skills. Problem solving was enhanced by the MCI experience, since learning took place whilst victims were prioritised on the disaster scene.

Action two, to promote *partnership appreciation*, was evident in more than one of the critical outcomes. One of the students indicated that the trained group leader ‘directed what needed to be done’, everyone was ‘put at ease’ and the group leader helped to ‘organise the whole group’. This gratitude towards a paramedic supported the critical outcome of communicating effectively. A study done by Melby (2000:638) on the experiential learning experience of nursing students in emergency-care settings confirms that an appreciation of the skills and abilities of paramedics forms the foundation for enhanced inter-professional collaboration in this setting. It can thus be accepted that the experiences gained by the UFS students regarding collaborative appreciation will also promote their understanding of the critical outcome where they see the health sciences world as part of a set of related systems. The quantitative data supported this aspect, since a positive attitude change towards role-players was experienced by 55% of the students.

The continuous reflective debriefing to *improve student coping mechanisms* (action 3) is represented by the intermingled AR cycles on the diagonal line of the model (Figure 1, number 5) referred to as ‘formative reflection’ by Seale *et al.* (2005:216). Seale *et al.* (2005:216) argue that reflection processes contribute to both the quality improvement of the project involved and to knowledge production (Figure 1, number 6). A number of students experienced the debriefing as empowering and felt that they had learnt what had gone wrong and what had gone right (Table 4). During the debriefing and reflection sessions with the students, reflective recommendations for improvement were made (Table 5). These recommendations were implemented during AR set two. The effect of these recommendations as seen by the facilitators is documented in Table 5. Students suggested that mock injuries (moulage) would be more realistic. Upon reflection, the facilitators felt that the authenticity of the MCI improved and resulted in quality. Additional information sessions, with real-life scenarios, were arranged for the students before the next MCI. The facilitators agreed that the application and integration of knowledge improved, which may result in critical thinking and deep learning.

**TABLE 5:** Reflective recommendations made in action research set one and the effect of its implementation in action research set two.

Reflective student recommendations: AR set one	Facilitator reflection on implemented recommendations: AR set two
Provide sufficient MCI information to optimise learning experience	Application and integration of knowledge improved
Simulation of real blood and injuries to be more realistic	Authenticity of the MCI improved
Prompt logistical arrangements and briefing should be done	Effect of proper arrangements before the MCI was noticeable to the facilitators
Make sure that adequate equipment is available	Not always possible in MCI
Be organised in order to start when planned	Better organised, but still did not start on time
Better co-operation between students and partners should take place	Orientation session contributed to better co-operation
Nurses need more training in emergency care	Implementation in hospital was a valuable learning experience

AR, action research; MCI, mass casualty incident.

Working in smaller groups to *increase student exposure*, each with a paramedic group leader (action 4), exposed more students to the MCI activity. Students’ comments on effective communication such as the delegation of tasks and the coordination of actions testify to the value of the leader in the smaller groups (Table 4). It is noteworthy to point out that many of the problems faced in disasters are not caused by shortcomings in medical competence but stem from failures in leadership and management (Alexander, Bandiera & Mazurik 2005:40). As a recommendation, the critical cross-field outcome to manage self is an important aspect to focus on in future incidents, as the students realised that they should stay focused, be patient and not panic (Table 4).

#### Reflection on actions research set one

AR set one is represented by one step of the step-up model. As mentioned previously, each step represents an AR set, where each set consists of two phases, namely a reflection phase (Figure 1, number 4) and an action phase (Figure 1, number 3). Formative reflection, indicated by the intermingled AR circles (Figure 1, number 5), occurred throughout the action phase and involved all the faculty members, with only occasional involvement of the other partners.

Summative reflection on the results took place annually at the reflection points (Figure 1, number 1). Seale **et al.** (2005:216) explain that the planning point (Figure 1, number 2) provides a basis for the identification and implementation of actions. This planning point extends the reflection process and leads to knowledge production and innovative redesign. The forward movement to the right of the reflection phase indicates the contribution to knowledge creation (Figure 1, number 6), such as this scholarly article. The upward movement of the action phase represents the quality improvement of the MCI and student learning.

## Background of actions research set two

When AR set two commenced, the MCI was implemented as previously in the field, but this time it entailed a stampede during a soccer match. At the same time, an additional MCI took place in a hospital-based environment in the big trauma unit of one of the provincial hospitals to promote student learning related to trauma in a hospital setting. The hospital’s trauma coordinator welcomed the idea as he wanted to test the readiness of the new trauma unit.

The student questionnaire used for AR set one was reused for AR set two, but with the addition of questions pertaining to the MCI in the hospital. The roles were expanded where the trauma nurse and doctor were included. A briefing session related to the complex setup in the hospital before the MCI was included in the preparation of the students.

### Brief discussion of actions research set two

The full set of results related to AR set two will not be discussed here. The focus of this set was on the trauma unit in the hospital setting. Mention will be made only of the results of the additional questions (Table 6). As shown in Table 6, 40% of the students found the briefing before the MCI helpful. The value of a trauma nurse is referred to by one of the students, who described their ability to ‘act fast and thoroughly so that lives can be saved’. The briefing before the MCI promoted the managing of self (Table 6). Students commented on aspects such as confidence, and an ability to prioritise and act fast.

**TABLE 6:** Results of action research set two according to the critical cross-field outcomes (shortened version).

Critical cross-field outcomes	Student questionnaire results (*N *= 45)	Percentage	Results all partners (qualitative direct quotes related to critical outcomes)
Seeing world as a set of related systems	Clarity of roles		
	Trauma nurse	64	‘act fast and thoroughly so that lives can be saved’
	Trauma doctor	67	‘to be organised and capable’
Managing of self	Effect of briefing before action of the MCI	40	‘to have confidence’
			‘to prioritise’
			‘to be prepared at all times’
			‘do not panic’
			‘act fast’
			‘time management is crucial in patient resuscitation’
			‘to have patience’
			‘time is valuable’

MCI, mass casualty incident.

Other references to the group leader’s characteristics were expressed as ‘guidance’, ‘instruction’ and ‘responsibility’. The added role-players (trauma nurse and trauma doctor) were described as ‘organised’ and ‘capable’. Uys and Gwele (2005:218) state that collaboration from various professions provides rich learning experiences. In this case, the students saw how the individual activities of the various role-players came together to serve the greater purpose and thus promote full understanding.

## Conclusion

The value of the research done on the simulated MCIs contexts described above is twofold. In view of the analysis of the collected data the researchers concluded that the focus on a combination of life skills (critical cross-field outcomes) and programme outcomes had a positive impact on the attitudes of students towards the partners. Quality emergency training thus results in a positive attitude between partners, with possible better real-life collaboration in various emergency contexts. One can conclude, furthermore, that the implementation of the critical outcomes was promoted during the MCI.

Secondly, the step-up AR model, as a method of enquiry, provided a structured framework to describe how it was used to improve the quality of trauma-related nursing educational practice, within a nursing programme. The step-up AR model proved to be a collaborative process that improves educational practice. The action phase contributed to the quality of the emergency care programme and the reflection phase contributed to the creation of knowledge of the discipline. One of the facilitators of the students made the following significant comment: ‘The step-up model provided structure and rigour to the research process, but allowed for flexibility during the implementation which would not be possible with traditional research.’ To improve the quality and rigour of educational programmes, it is therefore recommended that the step-up AR model be routinely used in the execution of educational practices.

This Web address below links to a video clip of the MCI: <http://www.youtube.com/watch?v=6sWDm9jE0S4>

## Footnotes

1 Critical cross-field outcomes are the broad, cross-curricular outcomes that underpin the learning process in all its facets (Van der Horst & Mc Donald 2005; Vasuthevan & Viljoen 2003). These outcomes are given legal form as generic skills for all teaching and learning, as stated in the *South African Qualifications Authority Act* (1995).
